# Fixed Point Results of Locally Contractive Mappings in Ordered Quasi-Partial Metric Spaces

**DOI:** 10.1155/2013/194897

**Published:** 2013-08-25

**Authors:** Abdullah Shoaib, Muhammad Arshad, Jamshaid Ahmad

**Affiliations:** ^1^Department of Mathematics, International Islamic University, H-10, Islamabad 44000, Pakistan; ^2^Department of Mathematics, COMSATS Institute of Information Technology, Chak Shahzad, Islamabad 44000, Pakistan

## Abstract

Fixed point results for a self-map satisfying locally contractive conditions on a closed ball in an ordered 0-complete quasi-partial metric space have been established. Instead of monotone mapping, the notion of dominated mappings is applied. We have used weaker metric, weaker contractive conditions, and weaker restrictions to obtain unique fixed points. An example is given which shows that how this result can be used when the corresponding results cannot. Our results generalize, extend, and improve several well-known conventional results.

## 1. Introduction and Preliminaries

Fixed points results of mappings satisfying certain contractive conditions on the entire domain have been at the centre of vigorous research activity, (see [1–3]) and it has a wide range of applications in different areas such as nonlinear and adaptive control systems, parameterize estimation problems, fractal image decoding, computing magnetostatic fields in a nonlinear medium, and convergence of recurrent networks (see [4–6]).

Recently, many results appeared related to fixed point theorem in complete metric spaces endowed with a partial ordering in the literature. Ran and Reurings [7] proved an analogue of Banach's fixed point theorem in metric space endowed with a partial order and gave applications to matrix equations. In this way, they weakened the usual contraction condition. Subsequently, Nieto and Rodríguez-López [8] extended the result in [7] for nondecreasing mappings and applied it to obtain a unique solution for a 1st-order ordinary differential equation with periodic boundary conditions. Thereafter, many works related to fixed point problems have also been considered in partially ordered metric spaces (see [7–11]).

On the other hand notion of a partial metric space was introduced by Matthews in [12]. In partial metric spaces, the distance of a point from itself may not be zero. Partial metric spaces have applications in theoretical computer science (see [13]). Altun and Erduran [14] and Paesano and Vetro [15] used the idea of partial metric space and partial order and gave some fixed point theorems for contractive condition on ordered partial metric spaces. Recently, Karapınar et al. [16] introduced the concept of quasi-partial metric space. Romaguera [17] has given the idea of 0-complete partial metric space. Nashine et al. [18] used this concept and proved some classical results.

From the application point of view the situation is not yet completely satisfactory because it frequently happens that a mapping *T* is a contraction not on the entire space *X* but merely on a subset *Y* of *X*. However, if *Y* is closed and a sequence {*x*_*n*_} in *X* converges to some *x* in *X*, then by imposing a subtle restriction on the choice of *x*_0_, one may force the sequence to stay eventually in *Y*. In this case, one can establish the existence of a fixed point of *T*. Arshad et al. [19] proved a significant result concerning the existence of fixed points of a mapping satisfying a contractive conditions on closed ball in a complete dislocated metric space. Other results on closed ball can be seen in [20, 21]. In this paper we have obtained fixed point theorems for dominated self-mappings in a 0-complete ordered quasi-partial metric space on closed ball under several contractive conditions to generalize, extend and improve some classical fixed point results. We have used weaker contractive condition and weaker restrictions to obtain unique fixed point. Our results do not exist even yet in metric spaces. An example shows how this result can be used when the corresponding results cannot.

Consistent with [16, 22, 23] the following definitions and results will be needed in the sequel.

**Definition 1 (see [16])**

A quasi-partial metric is a function *q* : *X* × *X* → *R* ^+^ satisfying the following:

- if 0 ≤ *q*(*x*, *x*) = *q*(*x*, *y*) = *q*(*y*, *y*), then *x* = *y* (equality);
- *q*(*x*, *x*) ≤ *q*(*y*, *x*) (small self-distances);
- *q*(*x*, *x*) ≤ *q*(*x*, *y*) (small self-distances);
- *q*(*x*, *z*) + *q*(*y*, *y*) ≤ *q*(*x*, *y*) + *q*(*y*, *z*) (triangle inequality), for all *x*, *y*, *z* ∈ *X*.

The pair (*X*, *q*) is called a quasi-partial metric space.

Note that if *q*(*x*, *y*) = *q*(*y*, *x*) for all *x*, *y* ∈ *X*, then (*X*, *q*) becomes a partial metric space (*X*, *p*). Moreover if *q*(*x*, *x*) = 0 for all *x* ∈ *X*, then (*X*, *q*) and (*X*, *p*) become a quasimetric space and a metric space, respectively. Also *p*_*q*_(*x*, *y*) = (1/2)[*q*(*x*, *y*) + *q*(*y*, *x*)],  *x*, *y* ∈ *X* is a partial metric on *X*. The function *d*_*p*_*q*__ : *X* × *X* → *R* ^+^ defined by *d*_*p*_*q*__(*x*, *y*) = *q*(*x*, *y*) + *q*(*y*, *x*) − *q*(*x*, *x*) − *q*(*y*, *y*) is a (usual) metric on *X*. The ball $\bar{B(x,\varepsilon )}$, where $\bar{B(x,\varepsilon )}=\{y\in X:q(x,y)\leq \varepsilon +q(x,x)\}$, is a closed ball in quasi-partial metric space, for some *x* ∈ *X* and *ε* > 0.

**Definition 2 (see [16])**

Let (*X*, *q*) be a quasi-partial metric. Then, we have the following;

- A sequence {*x*_*n*_} in (*X*, *q*) converges to a point *x* ∈ *X* if and only if lim⁡_*n*→*∞*_⁡  *q*(*x*, *x*_*n*_) = lim⁡_*n*→*∞*_⁡  *q*(*x*_*n*_, *x*) = *q*(*x*, *x*).
- A sequence {*x*_*n*_} in (*X*, *q*) is called a Cauchy sequence if the limits lim⁡_*n*,*m*→*∞*_⁡  *q*(*x*_*n*_, *x*_*m*_) and lim⁡_*n*,*m*→*∞*_⁡  *q*(*x*_*m*_, *x*_*n*_) exist (and are finite).
- The space (*X*, *q*) is said to be complete if every Cauchy sequence {*x*_*n*_} in (*X*, *q*) converges to a point *x* ∈ *X* such that (*x*, *x*) = lim⁡_*n*,*m*→*∞*_⁡  *q*(*x*_*n*_, *x*_*m*_) = lim⁡_*n*,*m*→*∞*_⁡  *q*(*x*_*m*_, *x*_*n*_).

**Lemma 3 (see [16])**

Let (*X*, *q*) be a quasi-partial metric space, let (*X*, *p*_*q*_) be the corresponding partial metric space, and let (*X*, *d*_*p*_*q*__) be the corresponding metric space. These statements are equivalent. (i) The sequence {*x*_*n*_} is Cauchy in (*X*, *q*). (ii) The sequence {*x*_*n*_} is Cauchy in (*X*, *p*_*q*_). (iii) The sequence {*x*_*n*_} is Cauchy in (*X*, *d*_*p*_*q*__). These statements are also equivalent. (i) (*X*, *q*) is complete. (ii) (*X*, *p*_*q*_) is complete. (iii) (*X*, *d*_*p*_*q*__) is complete.

**Definition 4**

Let (*X*, ⪯) be a partial ordered set. Then *x*, *y* ∈ *X* are called comparable if *x*⪯*y* or *y*⪯*x* holds.

**Definition 5 (see [22])**

Let (*X*, ⪯) be a partially ordered set. A self-mapping *f* on *X* is called dominated if *fx*⪯*x* for each *x* in *X*.

**Example 6 (see [22])**

Let *X* = [0,1] be endowed with the usual ordering and *f* : *X* → *X* defined by *fx* = *x* ^*n*^ for some *n* ∈ *ℕ*. Since *fx* = *x* ^*n*^ ≤ *x* for all *x* ∈ *X*, therefore *f* is a dominated map.

**Theorem 7 (see [23])**

Let (*X*, *d*) be a complete metric space, *S* : *X* → *X* a mapping, *r* > 0, and *x*_0_ an arbitrary point in *X*. Suppose there exists *k* ∈ [0,1) with

(1) $$\begin{array}{c} d\left(Sx,Sy\right)\leq kd\left(x,y\right),\;for\;\;all\;x,y\in Y=\bar{B\left(x_{0},r\right)} \end{array}$$

and *d*(*x*_0_, *Sx*_0_)<(1 − *k*)*r*. Then there exists a unique point x* in $\bar{B(x_{0},r)}$ such that x* = Sx*.

## 2. Fixed Points of Dominated Mapping

In this section we introduce the notion of a 0-complete quasi-partial metric spaces. We also prove some results in these spaces.

**Definition 8**

Let (*X*, *q*) be a quasi-partial metric space.

- A sequence {*x*_*n*_} in (*X*, *q*) is called 0-Cauchy if lim⁡_*n*,*m*→*∞*_⁡  *q*(*x*_*n*_, *x*_*m*_) = 0 or lim⁡_*n*,*m*→*∞*_ *q*(*x*_*m*_, *x*_*n*_) = 0.
- The space (*X*, *q*) is called 0-complete if every 0-Cauchy sequence in *X* converges to a point *x* ∈ *X* such that *q*(*x*, *x*) = 0.

It is easy to see that every 0-Cauchy sequence in (*X*, *q*) is Cauchy in (*X*, *d*_*p*_*q*__) and if (*X*, *q*) is complete, then it is 0-complete but the converse assertions do not hold. For example, the space *X* = [0, +*∞*)∩*Q* with *q*(*x*, *y*) = |*x* − *y* | +|*x*| is a 0-complete quasi-partial metric space but it is not complete (since *d*_*p*_*q*__(*x*, *y*) = 2 | *x* − *y*| and (*X*, *d*_*p*_*q*__) is not complete).

Moreover, if (*X*, *d*) is complete quasimetric space which is also quasi-partial, then it is 0-complete, quasi-partial metric space.

**Definition 9**

Let *X* be a nonempty set. Then (*X*, ⪯, *q*) is called an ordered quasi-partial metric space if (i) *q* is a quasi-partial metric on *X* and (ii) ⪯ is a partial order on *X*.

**Theorem 10**

Let (*X*, ⪯, *q*) be a 0-complete ordered quasi-partial metric space, *S* : *X* → *X* a dominated map, and *x*_0_ an arbitrary point in *X*. Suppose that there exists *k* ∈ [0,1) such that

(2) q(Sx,Sy)≤kq(x,y)   for  all  comparable  elements  x,y  in  B(x0,r)¯.

And

(3) $$\begin{array}{c} q\left(x_{0},Sx_{0}\right)\leq \left(1-k\right)\left[r+q\left(x_{0},x_{0}\right)\right]. \end{array}$$

If, for a nonincreasing sequence {*x*_*n*_} in $\bar{B(x_{0},r)}$,  {*x*_*n*_} → *u* implies that *u*⪯*x*_*n*_, then there exists a point x* in $\bar{B(x_{0},r)}$ such that x* = Sx* and *q*(x*, x*) = 0. Moreover, x* is unique, if for every pair of elements *x*, *y* in *X*  there exists a point *z* ∈ *X* such that *z*⪯*x* and *z*⪯*y*.

**Proof**

Consider a Picard sequence *x*_*n*+1_ = *Sx*_*n*_ with initial guess *x*_0_, as *x*_*n*+1_ = *Sx*_*n*_⪯*x*_*n*_ for all *n* ∈ {0} ∪ *N*. We will prove that $x_{n}\in \bar{B(x_{0},r)}$ for all *n* ∈ *N* by mathematical induction. By using inequality (3), we have

(4) q(x0,x1)≤(1−k)[r+q(x0,x0)]≤r+q(x0,x0).

Therefore $x_{1}\in \bar{B(x_{0},r)}$. Now let $x_{2},\ldots ,x_{j}\in \bar{B(x_{0},r)}$ for some *j* ∈ *N*. As *x*_*n*+1_⪯*x*_*n*_, so using inequality (2), we obtain

(5) $$\begin{array}{c} q\left(x_{j},x_{j+1}\right)=q\left(Sx_{j-1},Sx_{j}\right)\leq kq\left(x_{j-1},x_{j}\right), \end{array}$$

which implies that,

(6) $$\begin{array}{c} q\left(x_{j},x_{j+1}\right)\leq k^{2}q\left(x_{j-2},x_{j-1}\right)\leq \cdots \leq k^{j}q\left(x_{0},x_{1}\right). \end{array}$$

Now

(7) q(x0,xj+1)≤q(x0,x1)+⋯+q(xj,xj+1) −[q(x1,x1)+⋯+q(xj,xj)]≤q(x0,x1)[1+⋯+kj−1+kj](by  inequality (6)),q(x0,xj+1)≤(1−k)[r+q(x0,x0)](1−kj+1)1−k(by  inequality (3)).

Thus $x_{j+1}\in \bar{B(x_{0},r).}$ Hence $x_{n}\in \bar{B(x_{0},r)}$ for all *n* ∈ *N*. Also *x*_*n*+1_⪯*x*_*n*_ for all *n* ∈ *N*. It implies that

(8) $$\begin{array}{c} q\left(x_{n},x_{n+1}\right)\leq k^{n}q\left(x_{0},x_{1}\right)\;\text{for}\;\text{all}\;n\in N. \end{array}$$

It follows that

(9) q(xn,xn+i)≤q(xn,xn+1)+⋯+q(xn+i−1,xn+i)≤knq(x0,x1)[1+⋯+ki−2+ki−1]→0  as  n→∞.

Notice that the sequence {*x*_*n*_} is a 0-Cauchy sequence in $\left(\bar{B(x_{0},r)},q\right)$. As $\bar{B(x_{0},r)}$ is closed so is 0-complete. Therefore there exists a point $x^{*}\in \bar{B(x_{0},r)}$ with

(10) $$\begin{array}{c} q\left(x^{*},x^{*}\right)=\underset{n\to \infty }{\lim}q\left(x_{n},x^{*}\right)=\underset{n\to \infty }{\lim}\;q\left(x^{*},x_{n}\right)=0. \end{array}$$

Now,

(11) $$\begin{array}{c} q\left(x^{*},Sx^{*}\right)\leq q\left(x^{*},x_{n}\right)+q\left(Sx_{n-1},Sx^{*}\right)-q\left(x_{n},x_{n}\right). \end{array}$$

On taking limit as *n* → *∞* and using the fact that x*⪯*x*_*n*_⪯*x*_*n*−1_, when *x*_*n*_ → x*, we have,

(12) $$\begin{array}{c} q\left(x^{*},Sx^{*}\right)\leq \underset{n\to \infty }{\lim}\left[q\left(x^{*},x_{n}\right)+kq\left(x_{n-1},x^{*}\right)\right]. \end{array}$$

Then by inequality (10), we have

(13) $$\begin{array}{c} q\left(x^{*},Sx^{*}\right)\leq 0. \end{array}$$

Similarly,

(14) $$\begin{array}{c} q\left(Sx^{*},x^{*}\right)\leq 0, \end{array}$$

and hence x* = Sx*.

*Uniqueness*. Let *y* be another point in $\bar{B(x_{0},r)}$ such that *y* = *Sy*. If x* and *y* are comparable, then

(15) $$\begin{array}{c} q\left(x^{*},y\right)=q\left(Sx^{*},Sy\right)\leq kq\left(x^{*},y\right). \end{array}$$

This shows that x* = *y*. Now if x* and *y* are not comparable then there exists a point $z\in \bar{B(x_{0},r)}$ which is lower bound of both x* and *y*, that is, *z*⪯x* and *z*⪯*y*. Moreover by assumption *z*⪯x*⪯*x*_*n*_ ⋯ ⪯*x*_0_. Now we will prove that $S^{n}z\in \bar{B(x_{0},r)}$:

(16) q(x0,Sz)≤q(x0,x1)+q(x1,Sz)−q(x1,x1)≤(1−k)[r+q(x0,x0)]+kq(x0,z)    (by inequality (2) and inequality (3)),q(x0,Sz)≤(1−k)r+(1−k)q(x0,x0)+k[r+q(x0,x0)]=r−rk+q(x0,x0)−kq(x0,x0)+rk +kq(x0,x0)=r+q(x0,x0).

It follows that $Sz\in \bar{B(x_{0},r)}$. Now let $S^{j}z\in \bar{B(x_{0},r)}$

(17) q(x0,Sj+1z)≤q(x0,x1)+q(x1,Sj+1z)−q(x1,x1)≤(1−k)[r+q(x0,x0)]+kq(x0,Sjz)(by inequality (2) and inequality (3)),q(x0,Sj+1z)≤(1−k)r+(1−k)q(x0,x0)+k[r+q(x0,x0)]≤r−rk+q(x0,x0)−kq(x0,x0)+rk+kq(x0,x0)≤r+q(x0,x0).

It follows that $S^{j+1}z\in \bar{B(x_{0},r)}$. Hence $S^{n}z\in \bar{B(x_{0},r)}$ for all *n* ∈ *N*. Now as *S* is dominated, it follows that *S* ^*n*−1^ *z*⪯*S* ^*n*−2^ *z*⪯⋯⪯*z*⪯x* and *S* ^*n*−1^ *z*⪯*y* for all *n* ∈ *N*, which further implies *S* ^*n*−1^ *z*⪯*S* ^*n*^ x* and *S* ^*n*−1^ *z*⪯*S* ^*n*^ *y* for all *n* ∈ *N* as *S* ^*n*^ x* = x* and *S* ^*n*^ *y* = *y* for all *n* ∈ *N*:

(18) q(x∗,y)=q(Snx∗,Sny)≤q(Snx∗,Sn−1z)+q(Sn−1z,Sny) −q(Sn−1z,Sn−1z)≤kq(Sn−1x∗,Sn−2z)+kq(Sn−2z,Sn−1y)(∵  by  inequality (2))   ⋮≤kn−2q(x∗,Sz)+kn−2q(Sz,y)→0as  n→∞.

Hence x* = *y*.

**Example 11**

Let *X* = [0, +*∞*)∩*Q* be endowed with order *x*⪯*y* if *q*(*x*, *x*) ≤ *q*(*y*, *y*) and let *q* : *X* × *X* → *R* ^+^ be the 0-complete ordered quasi-partial metric on *X* defined by *q*(*x*, *y*) = max⁡{*y* − *x*, 0} + *x*. Define

(19) Sx={110xif  x∈[0,1]∩Xx−49if  x∈(1,∞)∩X.

Clearly, *S* is dominated mapping. Take *x*_0_ = 1/2,  *r* = 1/2; then $\bar{B(x_{0},r)}=\left[0,1\right]\cap X$; we have *q*(*x*_0_, *x*_0_) = 1/2,  *k* = 4/9 with

(20) (1−k)[r+q(x0,x0)]=(1−49)[12+12]=59,q(x0,Sx0)=q(12,S(12))=q(12,120)=12<59.

Also if *x*, *y* ∈ (1, *∞*)∩*X*, then,

(21) q(Sx,Sy)=max⁡{y−49−x+49,0}+x−49=max⁡{y−x,0}+x−49≥49[max⁡{y−x,0}+x]=kq(x,y).

So the contractive condition does not hold on *X* in each case.

Now if

(22) x,y∈B(x0,r)¯∩X,

then,

(23) q(Sx,Sy)=max⁡{110y−110x,0}+110x=110q(x,y)<49q(x,y).

Therefore, all the conditions of Theorem 13 are satisfied. Moreover, 0 is the unique fixed point of *S* and *q*(0, 0) = 0.

In Theorem 10, the condition (3) is imposed to restrict the condition (2) only for *x*, *y* in $\bar{B(x_{0},r)}$ and Example 11 explains the utility of this restriction. However, the following result relaxes the condition (3) but imposes the condition (2) for all comparable elements in the whole space *X*.

**Theorem 12**

Let (*X*, ⪯, *q*) be a 0-complete ordered quasi-partial metric space, *S* : *X* → *X* a dominated map, and *x*_0_ an arbitrary point in *X*. Suppose there exists *k* ∈ [0,1) with

(24) q(Sx,Sy)≤kq(x,y),  for  all  comparable  elements  x,y  in  X.

If, for a nonincreasing sequence {*x*_*n*_} in *X*,  {*x*_*n*_} → *u* implies that *u*⪯*x*_*n*_, then there exists a point x* in *X* such that x* = Sx* and *q*(x*, x*) = 0. Moreover, x* is unique, if for every pair of elements *x*, *y* in *X*, there exists a point *z* ∈ *X* such that *z*⪯*x* and *z*⪯*y*.

In Theorem 10, the existence of a lower bound and for a nonincreasing sequence {*x*_*n*_} in *X*,  {*x*_*n*_} → *u* implies that *u*⪯*x*_*n*_ are imposed to restrict the condition (2) only for comparable elements. However, the following result relaxes these conditions but imposes the condition (2) for all elements in $\bar{B(x_{0},r)}$.

**Theorem 13**

Let (*X*, *q*) be a 0-complete partial metric space, *S* : *X* → *X* a map, and *x*_0_ an arbitrary point in *X*. Suppose there exists *k* ∈ [0,1) with

(25) q(Sx,Sy)≤kq(x,y), for  all  elements  x,y  in  B(x0,r)¯,q(x0,Sx0)≤(1−k)[r+q(x0,x0)].

Then there exists a unique point x* in $\bar{B(x_{0},r)}$ such that x* = Sx*. Further *q*(x*, x*) = 0.

Metric version of Theorem 10 is given below.

**Theorem 14**

Let (*X*, ⪯, *d*) be a complete ordered metric space, *S* : *X* → *X* a dominated map, and *x*_0_ an arbitrary point in *X*. Suppose that there exists *k* ∈ [0,1) such that

(26) d(Sx,Sy)≤kd(x,y)    for  all  comparable  elements  x,y  in  B(x0,r)¯,  d(x0,Sx0)≤(1−k)r.

If, for a nonincreasing sequence {*x*_*n*_} in $\bar{B(x_{0},r)}$,  {*x*_*n*_} → *u* implies that *u*⪯*x*_*n*_, then there exists a point x* in $\bar{B(x_{0},r)}$ such that x* = Sx* and *q*(x*, x*) = 0. Moreover, x* is unique, if for every pair of elements *x*, *y* in *X* there exists a point *z* ∈ *X* such that *z*⪯*x* and *z*⪯*y*.

**Theorem 15**

Let (*X*, ⪯, *q*) be a 0-complete ordered quasi-partial metric space, *S* : *X* → *X* a dominated map, and *x*_0_ an arbitrary point in *X*. Suppose that there exists *b* ∈ [0, 1/2) such that

(27) $$\begin{array}{c} q\left(Sx,Sy\right)\leq b\left[q\left(x,Sx\right)+q\left(y,Sy\right)\right] \end{array}$$

for all comparable elements *x*, *y* in $\bar{B(x_{0},r)}$.

And

(28) $$\begin{array}{c} q\left(x_{0},Sx_{0}\right)\leq \left(1-k\right)\left[r+q\left(x_{0},x_{0}\right)\right], \end{array}$$

where *k* = *b*/(1 − *b*). If, for a nonincreasing sequence {*x*_*n*_} in $\bar{B(x_{0},r)}$,  {*x*_*n*_} → *u* implies that *u*⪯*x*_*n*_, then there exists a point x* in $\bar{B(x_{0},r)}$ such that x* = Sx* and *q*(x*, x*) = 0. Moreover, x* is unique, if for any two points *x*, *y* in $\bar{B(x_{0},r)}$ there exists a point $z\in \bar{B(x_{0},r)}$ such that *z*⪯*x* and *z*⪯*y*, and

(29) $$\begin{array}{c} q\left(x_{0},Sx_{0}\right)+q\left(z,Sz\right)\leq q\left(x_{0},z\right)+q\left(Sx_{0},Sz\right)\;for\;\;all\;z⪯Sx_{0}. \end{array}$$

**Proof**

Consider a Picard sequence *x*_*n*+1_ = *Sx*_*n*_ with initial guess *x*_0_, as *x*_*n*+1_ = *Sx*_*n*_⪯*x*_*n*_ for all *n* ∈ {0} ∪ *N*. We will prove that $x_{n}\in \bar{B(x_{0},r)}$ for all *n* ∈ *N* by mathematical induction. By using inequality (28), we have

(30) q(x0,x1)≤(1−k)[r+q(x0,x0)]≤r+q(x0,x0).

Therefore $x_{1}\in \bar{B(x_{0},r)}$. Now let $x_{2},\ldots ,x_{j}\in \bar{B(x_{0},r)}$ for some *j* ∈ *N*. As *x*_*j*+1_⪯*x*_*j*_, so using inequality (27), we obtain

(31) q(xj,xj+1)=q(Sxj−1,Sxj)≤b[q(xj−1,xj)+q(xj,xj+1)]≤kq(xj−1,xj),

which implies that

(32) $$\begin{array}{c} q\left(x_{j},x_{j+1}\right)\leq k^{2}q\left(x_{j-2},x_{j-1}\right)\leq \cdots \leq k^{j}q\left(x_{0},x_{1}\right). \end{array}$$

Now,

(33) q(x0,xj+1)≤q(x0,x1)+⋯+q(xj,xj+1) −[q(x1,x1)+⋯+q(xj,xj)]≤q(x0,x1)[1+⋯+kj−1+kj](by  inequality (32)),q(x0,xj+1)≤(1−k)[r+q(x0,x0)](1−kj+1)1−k(by  inequality (28)).

Thus $x_{j+1}\in \bar{B(x_{0},r)}$. Hence $x_{n}\in \bar{B(x_{0},r)}$ for all *n* ∈ *N*. Also *x*_*n*+1_⪯*x*_*n*_ for all *n* ∈ *N*. It implies that

(34) $$\begin{array}{c} q\left(x_{n},x_{n+1}\right)\leq k^{n}q\left(x_{0},x_{1}\right)\;\text{for}\;\text{all}\;n\in N. \end{array}$$

It follows that

(35) q(xn,xn+i)≤q(xn,xn+1)+⋯+q(xn+i−1,xn+i)q(xn,xn+i)≤knq(x0,x1)[1+⋯+ki−2+ki−1]→0  as  n→∞.

Notice that the sequence {*x*_*n*_} is a 0-Cauchy sequence in $\left(\bar{B(x_{0},r)},q\right)$. As $\bar{B(x_{0},r)}$ is closed, so is 0-complete. Therefore there exists a point $x^{*}\in \bar{B(x_{0},r)}$ with

(36) $$\begin{array}{c} q\left(x^{*},x^{*}\right)=\underset{n\to \infty }{\lim}\;q\left(x_{n},x^{*}\right)=\underset{n\to \infty }{\lim}\;q\left(x^{*},x_{n}\right)=0. \end{array}$$

Now,

(37) $$\begin{array}{c} q\left(x^{*},Sx^{*}\right)\leq q\left(x^{*},x_{n}\right)+q\left(Sx_{n-1},Sx^{*}\right)-q\left(x_{n},x_{n}\right). \end{array}$$

On taking limit as *n* → *∞* and using the fact that x*⪯*x*_*n*_⪯*x*_*n*−1_, when *x*_*n*_ → x*, we have,

(38) q(x∗,Sx∗)≤lim⁡n→∞[q(x∗,xn)+b{q(xn−1,Sxn−1)+q(x∗,Sx∗)}]≤lim⁡n→∞  b{kn−1q(x0,x1)+q(x∗,Sx∗)}≤lim⁡n→∞  knq(x0,x1)≤0.

Similarly,

(39) $$\begin{array}{c} q\left(Sx^{*},x^{*}\right)\leq 0, \end{array}$$

and hence x* = Sx*. Now,

(40) q(x∗,x∗)=q(Sx∗,Sx∗)≤b{q(x∗,Sx∗)+q(x∗,Sx∗)}(1−2b)q(x∗,x∗)≤0.

This implies that

(41) $$\begin{array}{c} q\left(x^{*},x^{*}\right)=0. \end{array}$$

*Uniqueness.* Let *y* be another point in $\bar{B(x_{0},r)}$ such that *y* = *Sy*. If x* and *y* are comparable, then

(42) q(x∗,y)=q(Sx∗,Sy)≤b[q(x∗,Sx∗)+q(y,Sy)]=b[q(x∗,x∗)+q(y,y)]=bq(y,y)≤q(y,y).

Using the fact that *q*(*y*, *y*) ≤ *q*(x*, *y*), we have *q*(x*, *y*) = 0. Similarly, *q*(*y*, x*) = 0. Now if x* and *y* are not comparable to then there exists a point $z\in \bar{B(x_{0},r)}$ which is a lower bound of both x* and *y*. Moreover by assumption *Sz*⪯*z*⪯x*⪯*x*_*n*_ ⋯ ⪯*x*_0_. Now by using inequality (27), we have

(43) q(Sx0,Sz)≤b[q(x0,x1)+q(z,Sz)]≤b[q(x0,z)+q(x1,Sz)](by inequality (29)),  q(x1,Sz)≤kq(x0,z).

(44) q(x0,Sz)≤q(x0,x1)+q(x1,Sz)−q(x1,x1)≤q(x0,x1)+kq(x0,z)   (by inequality (43)),q(x0,Sz)≤(1−k)[r+q(x0,x0)]+k[r+q(x0,x0)]=r+q(x0,x0).

It follows that $Sz\in \bar{B(x_{0},r)}$. Now, we will prove that $S^{n}z\in \bar{B(x_{0},r)}$, by using mathematical induction to apply inequality (27). Let $S^{2}z,\ldots ,S^{j}z\in \bar{B(x_{0},r)}$ for some *j* ∈ *N*. As *S* ^*j*^ *z*⪯*S* ^*j*−1^ *z*⪯⋯⪯*z*⪯x*⪯*x*_*n*_ ⋯ ⪯*x*_0_; then,

(45) q(Sjz,Sj+1z)=q(S(Sj−1z),S(Sjz))≤b[q(Sj−1z,Sjz)+q(Sjz,Sj+1z)],

which implies that

(46) q(Sjz,Sj+1z)≤kq(Sj−1z,Sjz)≤k2q(Sj−2z,Sj−1z)≤⋯≤kjq(z,Sz).

Now,

(47) q(xj+1,Sj+1z)=q(Sxj,S(Sjz))≤b[q(xj,Sxj)+q(Sjz,Sj+1z)]≤b[kjq(x0,x1)+kjq(z,Sz)](by inequality (32) and inequality (46))≤bkj[q(x0,z)+q(x1,Sz)](by inequality (29))≤bkj[q(x0,z)+kq(x0,z)]=kj+1q(x0,z0).

Now,

(48) q(x0,Sj+1z)≤q(x0,x1)+q(x1,x2)+⋯+q(xj,xj+1)+q(xj+1,Sj+1z)≤q(x0,x1)+kq(x0,x1)+⋯+kj+1q(x0,z),(by  inequality (32) and inequality (47))q(x0,Sj+1z)≤q(x0,x1)[1+k+⋯kj]+kj+1r,    (as  z∈B(x0,r)¯  )q(x0,Sj+1z)≤(1−k)r(1−kj+1)1−k+kj+1r=r.

It follows that $S^{j+1}z\in \bar{B(x_{0},r)}$ and hence $S^{n}z\in \bar{B(x_{0},r)}$. Now inequality (46) can be written as

(49) $$\begin{array}{c} q\left(S^{n}z,S^{n+1}z\right)\leq k^{n}q\left(z,Sz\right)\to 0\;\text{as}\;n\to \infty . \end{array}$$

Now,

(50) q(x∗,y)=q(Sx∗,Sy)≤q(Sx∗,Sn+1z)+q(Sn+1z,Sy)≤b[q(x∗,Sx∗)+q(Snz,Sn+1z)] +b[q(Snz,Sn+1z)+q(y,Sy)]≤bq(x∗,x∗)+2bq(Snz,Sn+1z)+bq(y,y)≤bq(y,y)≤q(y,y)(by  inequality (41) and inequality (49)).

Using the fact that *q*(*y*, *y*) ≤ *q*(x*, *y*), we have *q*(x*, *y*) = 0. Similarly, *q*(*y*, x*) = 0. Hence x* = *y*.

In Theorem 15, the conditions (29), the existence of a lower bound and for a nonincreasing sequence {*x*_*n*_} in *X*,  {*x*_*n*_} → *u* implies that *u*⪯*x*_*n*_ are imposed to restrict the condition (27) only for comparable elements. However, the following result relaxes these conditions but imposes the condition (27) for all elements in $\bar{B(x_{0},r)}$.

**Theorem 16**

Let (*X*, *d*_*q*_) be a complete dislocated quasimetric space, *S* : *X* → *X* a map, and *x*_0_ an arbitrary point in *X*. Suppose there exists *k* ∈ [0, 1/2) with

(51) $$\begin{array}{c} d_{q}\left(Sx,Sy\right)\leq k\left[d_{q}\left(x,Sx\right)+d_{q}\left(y,Sy\right)\right], \end{array}$$

for all elements *x*, *y* in $\bar{B(x_{0},r)}\;\;$and

(52) $$\begin{array}{c} d_{q}\left(x_{0},Sx_{0}\right)\leq \left(1-\theta \right)r, \end{array}$$

where *θ* = *k*/(1 − *k*). Then there exists a unique point x* in $\bar{B(x_{0},r)}$ such that x* = Sx* and *d*_*q*_(x*, x*) = 0.

In Theorem 10, the conditions (28) and (29) are imposed to restrict the condition (27) only for comparable elements. However, the following result relaxes these conditions but imposes the condition (27) for all elements in *X*.

**Theorem 17**

Let (*X*, ⪯, *q*) be a 0-complete ordered quasi-partial metric space and let *S* : *X* → *X* be a dominated map. Suppose that there exists *b* ∈ [0, 1/2) such that

(53) $$\begin{array}{c} q\left(Sx,Sy\right)\leq b\left[q\left(x,Sx\right)+q\left(y,\text{S}y\right)\right] \end{array}$$

for all comparable elements *x*, *y* in *X*. If, for a nonincreasing sequence {*x*_*n*_} in *X*,  {*x*_*n*_} → *u* implies that *u*⪯*x*_*n*_, then there exists a point x* in *X* such that x* = Sx* and *q*(x*, x*) = 0. Moreover, x* is unique, if for any two points *x*, *y* in $\bar{B(x_{0},r)}$ there exists a point $z\in \bar{B(x_{0},r)}$ such that *z*⪯*x* and *z*⪯*y*.

**Theorem 18**

Let (*X*, ⪯, *q*) be a 0-complete ordered quasi-partial metric space, *S* : *X* → *X* a dominated map, and *x*_0_ an arbitrary point in *X*. Suppose that there exists *c* ∈ [0, 1/2) such that

(54) $$\begin{array}{c} q\left(Sx,Sy\right)\leq c\left[q\left(x,Sy\right)+q\left(Sx,y\right)\right] \end{array}$$

for all comparable elements *x*, *y* in $\bar{B(x_{0},r)}$.

And

(55) $$\begin{array}{c} q\left(x_{0},Sx_{0}\right)\leq \left(1-k\right)\left[r+q\left(x_{0},x_{0}\right)\right], \end{array}$$

where *k* = *c*/(1 − *c*). If, for a nonincreasing sequence {*x*_*n*_} in $\bar{B(x_{0},r)}$,  {*x*_*n*_} → *u* implies that *u*⪯*x*_*n*_, then there exists a point x* in $\bar{B(x_{0},r)}$ such that x* = Sx*. Further *q*(x*, x*) = 0.

**Proof**

Consider a Picard sequence *x*_*n*+1_ = *Sx*_*n*_ with initial guess *x*_0_. As *x*_*n*+1_ = *Sx*_*n*_⪯*x*_*n*_ for all *n* ∈ {0} ∪ *N*. We will prove that $x_{n}\in \bar{B(x_{0},r)}$ for all *n* ∈ *N* by mathematical induction. By using inequality (55), we have

(56) $$\begin{array}{c} q\left(x_{0},x_{1}\right)\leq r+q\left(x_{0},x_{0}\right). \end{array}$$

Therefore $x_{1}\in \bar{B(x_{0},r)}$. Now let $x_{2},\ldots ,x_{j}\in \bar{B(x_{0},r)}$ for some *j* ∈ *N*. As *x*_*n*+1_⪯*x*_*n*_, so using inequality (54), we obtain

(57) q(xj,xj+1)≤c[q(xj−1,xj)+q(xj,xj+1)  −q(xj,xj)+q(xj,xj)]≤kq(xj−1,xj),

which implies that

(58) $$\begin{array}{c} q\left(x_{j},x_{j+1}\right)\leq k^{2}q\left(x_{j-2},x_{j-1}\right)\leq \cdots \leq k^{j}q\left(x_{0},x_{1}\right). \end{array}$$

Now

(59) q(x0,xj+1)≤q(x0,x1)+⋯+q(xj,xj+1)−[q(x1,x1)+⋯+q(xj,xj)]q(x0,xj+1)≤(1−k)[r+q(x0,x0)](1−kj+1)1−k  (by  inequality (55)).

Thus $x_{j+1}\in \bar{B(x_{0},r).}$ Hence $x_{n}\in \bar{B(x_{0},r)}$ for all *n* ∈ *N*. Also *x*_*n*+1_⪯*x*_*n*_ for all *n* ∈ *N*. It implies that

(60) $$\begin{array}{c} q\left(x_{n},x_{n+1}\right)\leq k^{n}q\left(x_{0},x_{1}\right)\;\text{for}\;\text{all}\;n\in N. \end{array}$$

It follows that

(61) q(xn,xn+i)≤q(xn,xn+1)+⋯+q(xn+i−1,xn+i)q(xn,xn+i)≤knq(x0,x1)[1+⋯+ki−2+ki−1]→0  as  n→∞.

Notice that the sequence {*x*_*n*_} is a 0-Cauchy sequence in $\left(\bar{B(x_{0},r)},q\right)$. As $\bar{B(x_{0},r)}$ is closed so is 0-complete. Therefore there exists a point $x^{*}\in \bar{B(x_{0},r)}$ with

(62) $$\begin{array}{c} q\left(x^{*},x^{*}\right)=\underset{n\to \infty }{\lim}\;q\left(x_{n},x^{*}\right)=\underset{n\to \infty }{\lim}q\left(x^{*},x_{n}\right)=0. \end{array}$$

Now,

(63) $$\begin{array}{c} q\left(x^{*},Sx^{*}\right)\leq q\left(x^{*},x_{n}\right)+q\left(Sx_{n-1},Sx^{*}\right)-q\left(x_{n},x_{n}\right). \end{array}$$

On taking limit as *n* → *∞* and using the fact that x*⪯*x*_*n*_⪯*x*_*n*−1_, when *x*_*n*_ → x*, we have

(64) q(x∗,Sx∗)≤lim⁡n→∞[q(x∗,xn)+c{q(xn−1,Sx∗)+q(xn,x∗)}≤lim⁡n→∞[c{q(xn−1,x∗)+q(x∗,Sx∗)−q(x∗,x∗)+q(xn,x∗)}](1−c)q(x∗,Sx∗)≤0 (by  inequality (62)).

Similarly,

(65) q(Sx∗,x∗)≤0

and hence x* = Sx*.

**Remark 19**

We can obtain the partial metric, quasi-metric, and metric version of all theorems which are still not present in the literature.
